# Sinus aspergilloma in rheumatoid arthritis before or during tumor necrosis factor-alpha antagonist therapy

**DOI:** 10.1186/ar2849

**Published:** 2009-11-03

**Authors:** Ariane Leboime, Jean-Marie Berthelot, Yannick Allanore, Lama Khalil-Kallouche, Philippe Herman, Philippe Orcel, Frédéric Lioté

**Affiliations:** 1 Fédération de Rhumatologie, Pôle Appareil Locomoteur (centre Viggo Petersen), Hôpital Lariboisière, Paris Diderot University, 2 rue Ambroise Paré, Paris 75010, France; 2 Service de Rhumatologie, Pôle Appareil Locomoteur, CHRU de Nantes, 1 place Alexis Ricordeau, Nantes 44000, France; 3 Service de Rhumatologie, Pôle Appareil Locomoteur, Hôpital Cochin, Paris Descartes University, 27 rue du Faubourg saint Jacques, Paris 75014, France; 4 Service d'ORL, Pôle Tête Et Cou, Hôpital Lariboisière, Paris Diderot University, 2 rue Ambroise Paré, Paris 75010, France

## Abstract

**Introduction:**

In 2008, the Food and Drugs Administration required manufacturers of TNFα antagonists to strengthen their warnings about the risk of serious fungal infections in patients with rheumatoid arthritis (RA). Sinus aspergilloma occurs occasionally in RA patients and can progress to invasive Aspergillus disease. The purpose of this study was to describe symptomatic sinus aspergilloma in RA patients treated with TNFα antagonists.

**Methods:**

Retrospective descriptive study of symptomatic cases of sinus aspergilloma in patients with RA followed in three French university hospitals. A systematic literature review was performed.

**Results:**

Among 550 RA patients treated with TNFα antagonists, six (1.1%) had symptomatic maxillary aspergilloma diagnosed by computed tomography before or during TNFα antagonist therapy. None had chronic neutropenia. Aspergilloma treatment was with surgery only in all six patients. In the literature, we found 20 reports of Aspergillus infection in patients with chronic inflammatory joint diseases (including 10 with RA). Only 5/20 patients were treated with TNFα antagonists (invasive lung aspergillosis, n = 3; intracranial aspergillosis, n = 1; and sphenoidal sinusitis, n = 1).

**Conclusions:**

Otorhinolaryngological symptoms must be evaluated before starting or switching TNFα antagonists. Routine computed tomography of the sinuses before starting or switching TNFα antagonists may deserve consideration.

## Introduction

The risk of infection is increased in patients with rheumatoid arthritis (RA). Before the introduction of TNFα antagonists, a retrospective study showed a twofold increase in the risk of serious infections among RA patients compared with non-RA patients [1]. Factors that increase the risk of infections in RA include disease-related immune dysfunction (involving T cells such as T-helper type 1 cells and, as described more recently, T-helper type 17 cells) [2] and immunosuppressive effects of drugs used to treat the disease, such as long-term glucocorticoids, disease-modifying antirheumatic drugs (DMARDs), and TNFα antagonists [3,4]. Other factors may be involved, including immobility, skin breaks, joint surgery, leukopenia, diabetes mellitus, and chronic lung disease.

The infections encountered in RA patients affect a variety of sites (upper and lower respiratory tracts, lungs, joints, bone, skin, soft tissues, and so forth) [5] and can be caused by bacteria, viruses, fungi, or mycobacteria. RA patients may experience reactivation of latent infection such as tuberculosis, which is the most commonly reported granulomatous infection in patients treated with TNFα antagonists [6]. Preventive strategies have been developed to identify patients at risk for latent tuberculosis [7-9]. Other infections occurring during TNFα antagonist therapy include legionellosis, listeriosis, pneumocystosis, histoplasmosis, and aspergillosis [6,10]. A recent warning issued by the Food and Drugs Administration and supported by the American College of Rheumatology Drug Safety Committee draws attention to histoplasmosis and other invasive fungal infections, including fatal cases, reported in RA patients taking TNFα antagonists (FDA Alert 9/4/2008).

Among fungal infections, aspergillosis is usually due to *Aspergillus fumigatus *and produces a broad spectrum of presentations, ranging from benign allergic disease to invasive infection. Before starting TNFα antagonist therapy, a number of investigations are performed routinely to rule out contraindications such as infections. These investigations include a chest radiograph and a tuberculin skin test for evidence of tuberculosis, as well as other tests indicated by the clinical symptoms. Nasal and/or sinus symptoms (such as nasal obstruction, chronic rhinitis, postnasal drip, recurrent epistaxis, foul smell, facial pain or headache) should therefore be evaluated by computed tomography (CT) to look for sinus disorders, including sinus aspergilloma, despite the absence of epidemiological evidence that RA predisposes to patient-reported sinus disorders (allergic, viral or bacterial) [11]. Aspergilloma, also called fungus ball, is a clump of fungus growing in a cavity, in the lung or a sinus, often a maxillary sinus. Aspergilloma has been found in 3.7% of patients undergoing surgery for chronic inflammatory sinusitis [12].

Sinus aspergilloma is often asymptomatic and may therefore be overlooked during the workup performed before starting TNFα antagonist therapy. Furthermore, TNFα antagonists may exacerbate latent fungal infections, causing a focal aspergilloma to progress to invasive aspergillosis. Our objective was to investigate cases of sinus aspergilloma seen in RA patients before or during TNFα antagonist therapy. To this end, we conducted a retrospective study in three university hospitals and reviewed the relevant literature. The results suggest that routine CT of the sinuses may deserve consideration before starting TNFα antagonist therapy.

## Materials and methods

### Retrospective patient review

A retrospective descriptive study was carried out in three university hospitals. In France, TNFα antagonist therapy can be started only in hospital departments of internal medicine and rheumatology. Between 1999 and 2007, patients were identified using the database of each hospital and the keywords: (rheumatoid arthritis or spondylarthropathy) AND (aspergilloma or fungus ball).

Standardized forms were used to collect the following data: sex, age, disease duration, co-morbidities, symptomatic and immunosuppressive treatments received before the diagnosis of aspergilloma (including joint surgery), and otorhinolaryngological history. The clinical presentation and treatment of the aspergilloma were recorded. Since this was not a prospective study, no ethical approval has been considered. In addition, patient anonymity was preserved in all parts of the retrospective review and result presentation.

### Systematic literature review

We searched the PubMed database up to October 2008 and the abstracts of the EULAR and American College of Rheumatology scientific meetings held in 2005, 2006, 2007, and 2008. Two searches were carried out in the PubMed database, using the following keywords: (rheumatoid arthritis OR ankylosing spondylitis OR spondylarthritis) AND (aspergilloma OR fungus ball OR aspergillosis OR sinusitis). Case reports, case series, and reviews were selected and analyzed using a standard form.

## Results

### Patient identification

We identified six patients with sinus aspergilloma among 550 (6/550, 1.1%) patients with RA undergoing screening for, or receiving, TNFα antagonist therapy. Their distribution by study center was as follows: three out of 50 patients at the Lariboisière Hospital, Paris; two out of 200 patients at the Nantes Hospital, Nantes; and one out of 300 patients at the Cochin Hospital, Paris.

### Patient characteristics

The main patient characteristics are presented in Table 1. All six patients with aspergilloma were women meeting American College of Rheumatology criteria for RA [13]. The mean age (± standard deviation) was 58 ± 8 years and the mean RA duration was 20.0 ± 10.2 years. All six patients had severe joint destruction. Co-morbidities included hypertension in three patients and iron-deficiency anemia in two patients. Bronchiectasis was a feature in one patient. Two patients had a history of appropriately treated pulmonary tuberculosis, with no reactivation during TNFα antagonist therapy. None of the patients had diabetes mellitus.

**Table 1:** Characteristics of the six patients with rheumatoid arthritis and sinus aspergilloma

Case	RA duration^a ^(years)	Age at aspergilloma onset (years)	Co-morbidities	Previous RA treatment other than TNFα antagonists	TNFα antagonist, date	Surgery for RA
1	17	53	Hepatitis, hypertension, hypothyroidism, gastric ulcer, bronchiectasis	Glucocorticoid therapy, methotrexate, leflunomide	Infliximab, March 2003	No
2	32	66	Hypertension, tuberculosis, osteoporosis, uveitis, coronary artery disease	Glucocorticoid therapy, methotrexate	Infliximab, November 2001	Yes
3	15	47	Primary tuberculosis, iron-deficiency anemia	Glucocorticoid therapy, methotrexate, leflunomide, salazopyrine	Etanercept, February 2003	Yes
4	39	70	Hypertension	Glucocorticoid therapy, methotrexate	Infliximab, March 2002; etanercept, July 2003	No
5	15	53	--	Glucocorticoid therapy, methotrexate, salazopyrine, hydroxychloroquine	--	Yes
6	12	52	Chronic hepatitis B, iron-deficiency anemia, osteopenia	Glucocorticoid therapy, methotrexate, salazopyrine	--	Yes

^a^Rheumatoid arthritis (RA) duration at aspergilloma diagnosis.

Treatments for RA are also presented in Table 1. All patients had a history of inadequate disease control with glucocorticoids and methotrexate. Other DMARDs, including leflunomide, were used in two patients, one of whom was still on leflunomide at the time of aspergilloma diagnosis.

At the time of aspergilloma diagnosis, four patients were taking TNFα antagonist therapy (infliximab, n = 3; and etanercept, n = 1). Of these four patients, two were on methotrexate and one was on leflunomide; all four patients were on low-dose glucocorticoid therapy. None of the six patients had chronic neutropenia at the time of aspergilloma diagnosis. Four patients had a history of surgery on one or more joints.

### Description and treatment of the aspergillomas

**Figure 1:**
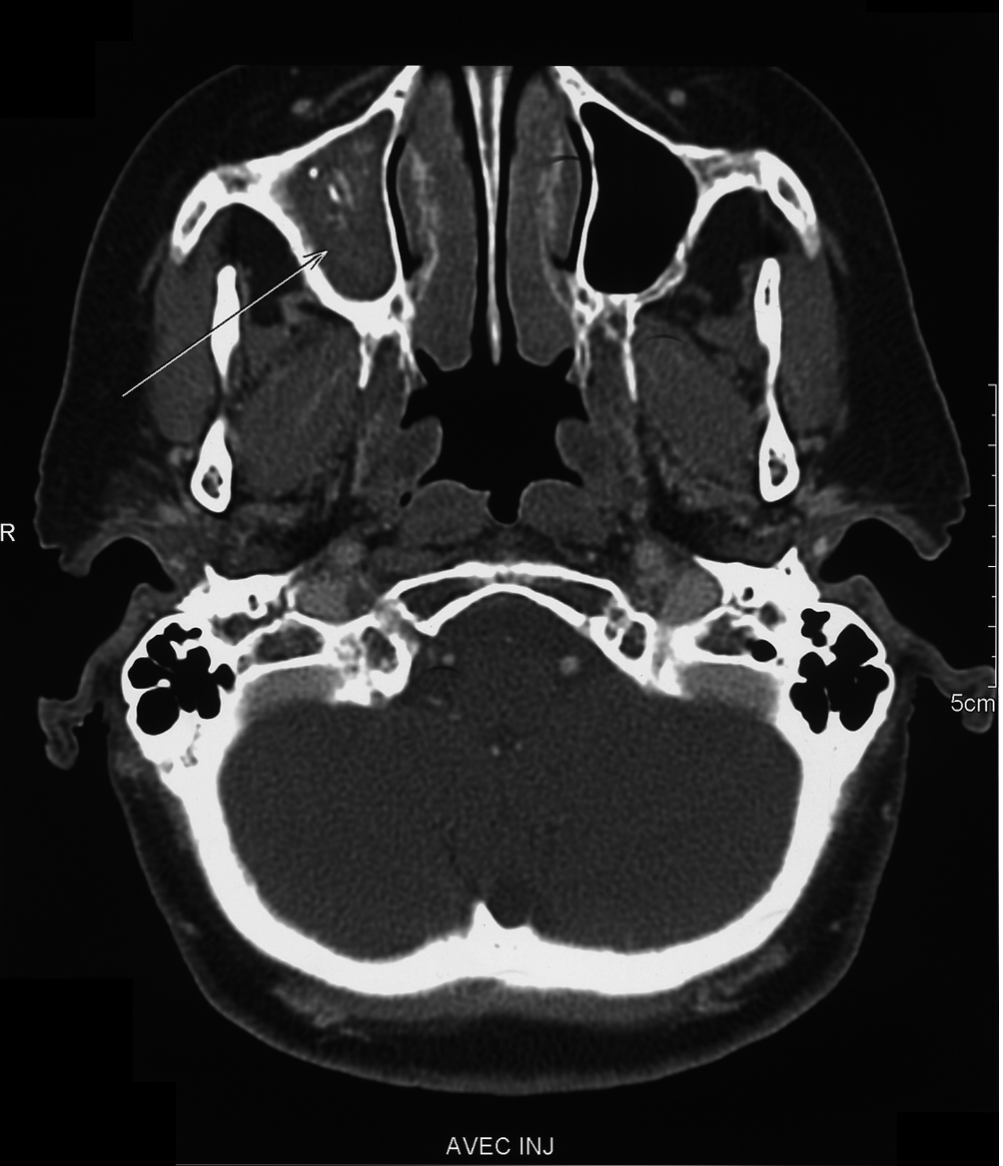
Aspergilloma visible as a soft tissue mass. Computed tomography (coronal view) of the maxillary sinus in Patient 6 before the first surgical procedure. Note the mass containing hyperdense foci that are highly suggestive of aspergilloma (arrow).

The main data are presented in Tables 2 and 3. A history of sinusitis was noted in four patients, including one patient who had had surgery for maxillary sinusitis. All six patients had unilateral aspergilloma located in a maxillary sinus. At diagnosis, all patients had symptoms such as nasal obstruction, recurrent sinusitis with facial pain, or hemorrhagic rhinorrhea. Serology for aspergillosis was negative in the three tested patients. For four patients, CT scans of the sinuses obtained before surgery were available as films or electronic files and were reviewed for bone involvement by an experienced otorhinolaryngology surgeon (PH). The aspergilloma was visible as a soft tissue mass (Figure 1). Hyperdense opacities were seen in three patients. In two patients the sinus wall was thickened, suggesting chronic inflammation.

**Table 2:** Previous otorhinolaryngological disease and aspergilloma characteristics

Case	Previous/active ENT disease	Maxillary sinus involved	Aspergilloma diagnosis	Rheumatoid arthritis treatment at aspergilloma diagnosis
1	Maxillary sinusitis treated surgically/active ENT symptoms	Right	December 2004	Infliximab, glucocorticoid therapy (8 mg/day), leflunomide (20 mg/day)
2	None/active ENT symptoms	Right	April 2007	Etanercept (25 mg/week), glucocorticoid therapy (6 mg/day), methotrexate (15 mg/day)
3	Chronic sinusitis/active ENT symptoms	Left	July 2005	Etanercept, glucocorticoid therapy
4	Chronic sinusitis/active ENT symptoms	Left	June 2007	Etanercept (50 mg/week), methotrexate (15 mg/day), glucocorticoid therapy (6 mg/day)
5	None/active ENT symptoms	Right	January 2005	Glucocorticoid therapy (7 mg/day), salazopyrine (1.5 g/day), methotrexate (15 mg/week)
6	Chronic sinusitis/active ENT symptoms	Right	March 2006	salazopyrine (2 g/day)

ENT, ear--nose--throat.

**Table 3:** Treatment of sinus aspergilloma and impact on TNFα antagonist therapy

Case	Systemic antifungal treatment	Surgical treatment	Impact on TNFα antagonist therapy
1	--	Aspergilloma removal after meatotomy in January 2005	Temporary discontinuation
2	--	May 2007	Treatment stopped before surgery
3	--	August 2005, September 2006	Temporary discontinuation
4	--	Aspergilloma removal by endoscopy in June 2007	Treatment stopped before surgery
5	--	Aspergilloma removal after meatotomy in January 2005	TNFα antagonist therapy considered contraindicated because of the aspergilloma
6	--	Aspergilloma removal after meatotomy in August 2006, December 2006, September 2007	TNFα antagonist therapy delayed for 18 months

The aspergilloma was removed surgically in all six patients. No systemic antifungal agents were given. Local aspergilloma recurrence developed in two patients and required one additional and two additional surgical procedures, respectively. The patient who had three surgical procedures in all experienced acute bleeding after the third operation, and required transfusion of a red cell pack and reoperation for hemostasis.

### Impact on TNFα antagonist therapy

As shown in Table 3, four patients discontinued TNFα antagonist therapy until surgery was performed. In one patient (Patient 6), TNFα antagonist therapy initiation was delayed because of the diagnosis of aspergilloma. One patient was therefore screened for but never received TNF antagonists.

### Review of the literature

We identified 23 cases of aspergillosis in patients with chronic inflammatory diseases. The underlying disease was RA in 12 patients (Patients 30 to 41) (Table 4), ankylosing spondylitis in nine patients (Patient 18 and Patients 42 to 49), chronic polyarthritis in one patient (Patient 50), and Crohn's disease in one patient (Patient 19) (Table 5).

**Table 4:** *Aspergillus *disease in rheumatoid arthritis patients: literature review

Type of Aspergillus disease	Site	TNFα antagonist	Other treatment	Outcome	Reference
Aspergilloma	Lung	No	Hydroxychloroquine	Death	[31]
Invasive aspergillosis	Lung	No	Methotrexate	Recovery	[32]
Aspergilloma	Lung	No	Glucocorticoid therapy	Recovery	[33]
Aspergilloma	Lung	No	Glucocorticoid therapy, salazopyrine, methotrexate	Recovery	[34]
Invasive aspergillosis	Lung	Infliximab	Glucocorticoid therapy, leflunomide	Recovery	[35]
Rheumatoid nodule colonization	Lung	No	Glucocorticoid therapy, methotrexate	Death	[36]
Aspergillosis	Lung	Infliximab	Methotrexate	?	[37]
Aspergillosis	Lung	Etanercept	Not available	?	[38]
Invasive aspergillosis	Lung	No	Not available	Not available	[39]
Aspergillosis	Lung	No	Glucocorticoid therapy, methotrexate, leflunomide	Death	[40]
Aspergillosis	Lung	No	Methotrexate	Recovery	[41]
Aspergilloma	Sphenoidal sinus	Infliximab	Glucocorticoid therapy	Recovery	[42]

**Table 5:** *Aspergillus *disease in patients with other chronic inflammatory joint diseases: literature review

Type of Aspergillus disease	Site	Joint disease	Other treatment^a^	TNFα antagonist	Outcome	Reference
Aspergilloma	Lung	Ankylosing spondylitis	?	--	?	[43]
Aspergilloma	Lung	Ankylosing spondylitis	Radiation therapy	--	Recovery	[44]
Aspergilloma	Lung	Ankylosing spondylitis	?	--	Recovery	[45]
Aspergilloma	Lung	Ankylosing spondylitis	?	--	?	[46]
Aspergilloma (× 2)	Lung	Ankylosing spondylitis	Radiation therapy	--	Recovery, recurrence	[47]
Aspergilloma + invasive aspergillosis	Lung	Ankylosing spondylitis	?	--	Improvement	[17]
Aspergilloma	Lung	Ankylosing spondylitis	?	?	Recovery	[48]
Aspergilloma	Lung	Ankylosing spondylitis	?	--	Recovery	[49]
Aspergillosis	Frontal sinus, meningitis, encephalitis	Chronic polyarthritis	GC	--	Recovery	[50]
Invasive aspergillosis	Lung	Crohn's disease	--	Infliximab	Death	[20]
Aspergillosis	Intra-cranial	Ankylosing spondylitis	GC	Etanercept	Recovery	[19]

^a^Including glucocorticoid therapy (GC) and disease-modifying antirheumatic drugs.

Of the 12 RA patients, four were receiving TNFα antagonist therapy (infliximab, n = 3; etanercept, n = 1) at diagnosis of aspergillosis. All three cases of lung aspergilloma in RA patients occurred during DMARD therapy without TNFα antagonist therapy. Of the three patients with invasive lung aspergillosis, one patient was on TNFα antagonist therapy. The four RA patients on TNFα antagonist therapy had severe Aspergillus disease; there were two cases of pulmonary aspergillosis, one case of invasive pulmonary aspergillosis, and one case of intracranial aspergillosis.

Of the nine patients with ankylosing spondylitis, one was on TNFα antagonist therapy and had a right orbital apex localization of aspergillosis. Eight patients had lung aspergilloma, and among them one progressed into an invasive lung aspergillosis. Interestingly, one patient with unclassified chronic polyarthritis had frontal sinus involvement and meningoencephalitis.

None of the case reports mentioned neutropenia at the time of diagnosis. All but two RA patients were taking glucocorticoids alone or with methotrexate (n = 4) or with leflunomide (n = 2, with methotrexate in one case). Little information was available about the treatments in the ankylosing spondylitis patients; however, two of these patients received radiation therapy to the spine. Of the 19 patients for whom outcome information was available, four (21%) patients died. Furthermore, one of the patients who recovered experienced a recurrence.

## Discussion

We describe the cases of six RA patients with symptomatic sinus aspergilloma diagnosed during screening for, or treatment with, TNFα antagonists. All six patients were treated surgically. This small series represents almost 1.1% of 550 RA patients treated with TNFα antagonists between 1999 and 2007 at three university hospitals in France.

The relatively high rate of sinus aspergilloma in our study was somewhat unexpected. A literature review, however, identified 64 cases of invasive aspergillosis in patients taking TNFα antagonists [10]. There were also 84 cases of invasive histoplasmosis and 64 cases of invasive candidiasis. The predominant clinical presentation of Aspergillus disease in this study of invasive fungal infections was invasive pulmonary aspergillosis. No cases of sinus involvement were noted, but no information was available about whether routine sinus imaging was performed [10]. In our study, CT of the sinuses was performed only to investigate symptoms. Sinus aspergilloma may remain asymptomatic for several years, however, and may therefore be underestimated.

Sinus involvement with Aspergillus may be either invasive or noninvasive. Invasive sinus aspergillosis may be indolent or fulminant. Noninvasive Aspergillus sinusitis may manifest as allergic fungal sinusitis or mycetoma (aspergilloma). Mucosal invasion by fungal hyphae and presence of a granulomatous response indicate invasive disease. However, the two forms may be difficult to differentiate; thus, allergic fungal sinusitis may spread intracranially and, on the other hand, indolent invasive aspergillosis may be well tolerated. Evaluating the risk of progression to invasive disease is crucial in patients with sinus aspergilloma. Noninvasive Aspergillus sinusitis, which usually remains confined to one sinus, occurs in immunocompetent patients; whereas invasive sinus aspergillosis chiefly affects immunocompromised patients, such as bone marrow transplant recipients or patients with prolonged neutropenia caused by chemotherapy [14] or hematologic malignancies.

Several cases of aspergilloma who progressed into an invasive form have been described. First, a fatal case of aspergilloma with progression to invasive disease after kidney transplantation and immunosuppressive treatment has been reported [15]. In a patient with diabetes mellitus and cirrhosis of the liver, a maxillary aspergilloma spread into the orbit and up to the cribriform plate, leading to the patient's death [16]. Elliott and colleagues described a patient with ankylosing spondylitis who had fever and cough. Diagnosis of concomitant aspergilloma and invasive aspergillosis of the lung was made by sputum analysis and histology of transbronchial lung biopsy. The patient improved with intraconazole treatment [17]. Finally, a patient with frontal sinus aspergilloma presented with right-sided pyocele expanding into the orbit; she had no detectable immunodeficiency [18]. Two of these cases [16,18] were unusually aggressive forms of noninvasive aspergilloma exhibiting tumor-like behavior with local spread and bone erosion but no histological invasion.

The extent to which drugs used to treat chronic inflammatory joint disease may promote progression of sinus aspergilloma to invasive aspergillosis deserves discussion. Little is known about the outcome of aspergilloma in patients taking TNFα antagonists. Our literature review identified a single previous case of sinus aspergilloma during TNFα antagonist therapy, in a patient with involvement of the sphenoidal sinus (Patient 38). Neither the mucosa nor the sinus wall was invaded in this patient. Of our six patients, four were on TNFα antagonist therapy at the time of aspergilloma diagnosis. None had bone erosions or spread to other sinuses. Our data suggest that the prognosis of maxillary sinus aspergilloma in RA patients on TNFα antagonist therapy may be similar to that in patients without RA or immunosuppressive treatment. Recurrences requiring repeat surgery occurred in two out of our six patients. Recurrences may complicate incomplete removal of aspergilloma during minimally invasive surgery, which is useful in these fragile patients but provides limited exposure.

We identified several previous reports of pulmonary aspergilloma and invasive aspergillosis in patients with chronic inflammatory joint diseases (Table 4). Another patient had central nervous system aspergillosis [19]. Most of these patients had RA or ankylosing spondylitis, although one patient had Crohn's disease [20]. All were taking immunosuppressive drugs such as glucocorticoids and methotrexate, and six patients were taking TNFα antagonists. No patient experienced progression from noninvasive to invasive Aspergillus disease after starting TNFα antagonist therapy. Anecdotal including invasive sphenoidal sinus aspergilloma and fatal cases have been reported in patients with vasculitis, such as Wegener's disease and temporal arteritis, but there are not included in the present review since anti-TNF agents are not indicated in these conditions.

Fungal cultures were positive in 80 to 97% of patients with chronic rhinosinusitis, and mycetoma was found in 13 to 28.5% of patients with chronic maxillary sinusitis [21,22]. *Aspergillus *spp. are the main pathogen found in chronic sinusitis (75%). *Candida albicans*, *Penicillium *spp., and *Streptomyces *spp. also occur [21]. *Aspergillus *spp. is a saprophytic ubiquitous fungus found in organic debris, dust, food, spices, and rotting plants. Of the nearly 200 species, only a few are pathogenic, predominantly *A. fumigatus*, *Aspergillus flavus*, and *Aspergillus niger*. Aspergillus is a filamentous fungus that has septate hyphae and reproduces as asexual conidia [16]. A higher incidence of Aspergillus disease has been reported in areas that have a hot dry climate - especially of *A. flavus*, often described in Sudan [23]. The fungus is usually acquired from an inanimate reservoir, by inhalation of airborne spores. Moreover, hospital construction work has been described as a risk factor for fungal infection [24]. Environmental measures such as impermeable barriers at construction sites, wearing face masks, and closing doors and windows should therefore be recommended in hospitals, especially in oncology or hematology units. Similar prophylactic measures may deserve consideration in hospitals managing patients on TNFα antagonist therapy; they have been adopted at our institution. Voriconazole prophylaxis has been found effective [25].

Ear nose and throat symptoms may escape the attention of rheumatologists during screening for, or treatment with, TNFα antagonist therapy or before switching from one TNFα antagonist to another. Patients should be asked routinely about ear nose and throat symptoms consistent with chronic sinus infection such as nasal obstruction, chronic rhinitis, postnasal drip, or foul smell. Facial pain or headache are perhaps more likely to be spontaneously reported by RA patients, who may ascribe these symptoms to their joint disease. Recurrent or refractory unilateral sinusitis should suggest bacterial superinfection of an aspergilloma. Although our patients were symptomatic at the time of aspergilloma diagnosis, sinus aspergilloma may remain asymptomatic in 13.2 to 20% of patients and may be diagnosed on imaging studies obtained for another reason [26,27].

CT is the investigation of choice for diagnosing sinus aspergilloma. Routine CT of the sinuses may therefore be advisable when screening patients for TNFα antagonist therapy, as well as to evaluate symptoms in patients already on TNFα antagonist therapy. The typical finding is partial or complete opacity of a sinus due to a soft tissue mass, usually in a maxillary sinus [28]. Hyperdense foci within the mass strongly suggest a mycetoma. The nature of these foci is unclear. Endodontic sealers may play a role, most notably those containing zinc oxide, which may promote the growth of Aspergillus by blocking the epithelial cilia [29]. Heavy metals such as iron and manganese may also produce calcification-like images. Bone sclerosis of the sinus wall is often described. Bone erosion is uncommon and may mistakenly suggest a tumor [30]. Bone erosion seems to be a reversible process caused by inflammation related to fungal growth and bacterial superinfection.

To our knowledge, there are no published controlled studies on the treatment of sinus aspergilloma. Patients with symptoms and specific CT scan findings should receive surgical treatment. In asymptomatic patients, it is unclear whether surgery should be deferred or performed immediately. Surgery involves a transnasal approach under endoscopic control, wide opening of the maxillary sinus (antrostomy), and removal of the entire aspergilloma. The inferior meatal or canine fossa approach may be used in combination with the transnasal approach. All six patients in our study underwent aspergilloma removal after antrostomy. Two patients required one or two additional procedures to treat recurrences.

## Conclusions

Aspergilloma is a noninvasive form of Aspergillus sinusitis encountered in immunocompetent patients. Progression to invasive aspergillosis may occur in patients with immune deficiencies caused by glucocorticoids, DMARDs, or TNFα antagonists. CT is the investigation of choice for diagnosing sinus aspergilloma and is inexpensive (€50 in France in 2008). CT of the sinuses might be considered in patients who are being screened for TNFα antagonist therapy, specifically those with ear nose and throat symptoms.

## Abbreviations

CT: computed tomography; DMARD: disease-modifying antirheumatic drug; RA: rheumatoid arthritis; TNF: tumor necrosis factor.

## Competing interests

The authors declare that they have no competing interests.

## Authors' contributions

FL conceived the study and helped to draft the manuscript. J-MB, YA, and PO helped with patient recruitment. PH helped to analyze the CT scan results. LK-K helped with patient recruitment. AL participated in drafting the study and performed the literature review. All authors read and approved the final manuscript.
